# Effects of CPAP withdrawal on sex hormone levels in patients with obstructive sleep apnoea: data from two randomised, controlled trials

**DOI:** 10.1016/j.sleepx.2026.100201

**Published:** 2026-09-11

**Authors:** Fabian A. Grewe, Sira Thiel, Sarah R. Haile, Mirko Peitzsch, Esther I. Schwarz, Noriane A. Sievi, Felix Beuschlein, Malcolm Kohler, Thomas Gaisl

**Affiliations:** a Department of Pulmonology, University Hospital Zurich, University of Zurich, Zurich, Switzerland; b Zollikerberg Hospital, Clinic of Internal Medicine, Zollikerberg, University of Zurich, Zurich, Switzerland; c Department of Epidemiology, Epidemiology, Biostatistics, and Prevention Institute, University of Zurich, Zurich, Switzerland; d Institute of Clinical Chemistry and Laboratory Medicine, University Hospital and Faculty of Medicine Carl Gustav Carus, Technical University Dresden, Dresden, Germany; e Department of Endocrinology and Diabetology, University Hospital Zurich, University of Zurich, Zurich, Switzerland; f The LOOP Zurich - Medical Research Center, Zurich, Switzerland; g Centre for Integrative Human Physiology, University of Zurich, Zurich, Switzerland

**Keywords:** Obstructive sleep apnoea, Continuous positive airway pressure, Sexual dysfunction, Sex hormones

## Abstract

Sexual dysfunction (SD) is common in patients with obstructive sleep apnoea (OSA). Although continuous positive airway pressure (CPAP) therapy improves SD, it does not consistently modify testosterone or estradiol levels, suggesting involvement of other hormonal pathways. We analysed sex hormone levels in 70 patients with OSA before and after two weeks of CPAP withdrawal. No statistically significant treatment effects were observed for testosterone, androstenedione, gestagens, dehydroepiandrosterone sulfate, luteinizing hormone or follicle-stimulating hormone, whereas dehydroepiandrosterone (DHEA) increased after CPAP withdrawal. As DHEA secretion is adrenocorticotropic hormone-responsive, this increase may point to stress-related mechanisms, rather than direct sex steroid-mediated pathways, as potential contributors to OSA-related SD.

## Highlights

• First study of a broad sex hormone panel and its response to CPAP withdrawal.
• CPAP withdrawal increased DHEA significantly.
• Most sex hormone levels remained stable after short-term CPAP withdrawal.
• The DHEA rise may reflect stress-related rather than gonadal mechanisms.

## 1 Introduction

Obstructive sleep apnoea (OSA) is a highly prevalent sleep-related breathing disorder [1]. Sexual dysfunction (SD) is common in patients with OSA and may manifest as reduced sexual desire, diminished sexual satisfaction, and impaired partner intimacy, in both women and men [2]. In addition, OSA may lead to decreased vaginal lubrication in women and erectile dysfunction in men [2]. Continuous Positive Airway Pressure (CPAP) has been shown to effectively treat OSA and short-term CPAP withdrawal is an established experimental model to investigate OSA pathophysiology [3]. Greater OSA severity, reflected by a higher apnoea–hypopnoea index (AHI), is associated with lower estradiol and testosterone levels; however, CPAP therapy does not consistently alter these concentrations, despite improving sexual quality of life in both women and men [[4], [5], [6]]. This discrepancy suggests that the beneficial effects of CPAP on sexual health may involve other hormonal pathways. However, knowledge on the influence of CPAP therapy on further sex hormones is mostly lacking. We therefore investigated whether CPAP treatment is associated with short-term changes in levels of sex hormones beyond estradiol and testosterone.

## 2 Materials and methods

### 2.1 Trial design

We analysed data from 70 patients (58 male and 12 female; 1 premenopausal and 11 postmenopausal) enrolled in two 2-week, parallel-group, randomised controlled trials, conducted in the University Hospital Zurich (Switzerland) [3,4]. The original studies were designed to assess whether CPAP withdrawal for 2 weeks affects cerebral vascular reactivity and exhaled breath patterns in patients with moderate-to-severe OSA. Participants were assigned to either sub-therapeutic CPAP (n = 33) or continuation of therapeutic CPAP (n = 37). Fasting blood samples were collected in the morning, at baseline and after two weeks (for more detailed information see Thiel et al. [3], Schwarz et al. [4]). Subjective daytime sleepiness was assessed using the Epworth Sleepiness Scale (ESS) in both parent trials [3,4]. The hormones evaluated in the current analysis included androstenedione, dehydroepiandrosterone (DHEA), 17α-hydroxyprogesterone, progesterone, total, bioavailable, and free testosterone, follicle-stimulating hormone (FSH) and luteinizing hormone (LH). These hormone outcomes were prespecified secondary endpoints of the parent trials. Dehydroepiandrosterone sulfate (DHEA-S) had also been measured and has additionally been analysed post-hoc. Levels of steroid hormones were analysed via liquid chromatography with tandem mass spectrometry. Treatment effects for the prespecified secondary endocrine endpoints were assessed using multivariable linear regression models, with adjustment for the corresponding baseline hormone level, parent study, sex, age, BMI, and the presence of hypertension. Free testosterone, testosterone sum, FSH and LH were not measured in one of the two parent trials [3], leading to structural missing data and should therefore be interpreted cautiously (see Supplementary Table 1). For the remaining prespecified hormones, only a small proportion of observations was missing and missing values were handled using multiple imputation by chained equations (MICE).

### 2.2 Participants

Eligibility criteria were at least one year of adherent CPAP therapy (>4 h daily, >80% of days), age between 20 and 75 years, apnoea–hypopnoea index (AHI) and/or 4% oxygen desaturation index (ODI4%) ≥20/h in an in-laboratory sleep study at the time of diagnosis, plus an ODI4% ≥15/h in a current nocturnal pulse oximetry after interrupting CPAP therapy for 4–5 nights. The trial was performed in accordance with the Good Clinical Practice guidelines. Further details have been reported previously and are summarised in the online supplement [5].

## 3 Results

The study period comprised February 2014 to December 2017. A study flow chart can be found in the previous publication [3]. Baseline characteristics (Table 1), including anthropometrics, comorbidities, OSA characteristics, and baseline sex hormone levels, did not differ between groups. No significant effects of CPAP withdrawal on the levels of testosterone, androstenedione, gestagens, FSH, LH, or DHEA-S were observed. DHEA-S showed a numerically greater increase in the CPAP-withdrawal group, but the adjusted treatment effect did not reach statistical significance in the exploratory complete-case analysis (p = 0.089). The only statistically significant effect of CPAP withdrawal in our multiple imputation analysis, after adjusting for the corresponding baseline DHEA level, parent study, sex, age, BMI, and the presence of hypertension, was a change in DHEA (mean change 0.86 ng/ml, 95% CI 0.17 to 1.56, p = 0.016) (Fig. 1). Overall, effect sizes were small. Higher diastolic office blood pressure was independently associated with greater DHEA levels, whereas no other cardiovascular parameters showed relevant associations.

**Table 1:** Baseline characteristics of the study participants, adapted and modified with permission from Thiel, S. et al., [5].

	Therapeutic CPAP (n = 37)	CPAP withdrawal (n = 33)
**Anthropometrics**		
Age, years	62.0 ± 10.6	60.9 ± 10.8
Sex, male (%)	31 (83.8%)	27 (81.8%)
Height, cm	174 ± 9	173 ± 8
Weight, kg	98 ± 19	104 ± 19
BMI, kg/m^2^	32.7 ± 6.5	34.6 ± 5.8
Neck circumference, cm	43.7 ± 3.8	43.6 ± 3.7
Waist circumference, cm	114.8 ± 13.1	117.6 ± 9.9
Hip circumference, cm	113.3 ± 11.6	117.9 ± 10.9
Waist:hip ratio	1.0 ± 0.1	1.0 ± 0.1
**Comorbidities**		
Active smoker, n (%)	4 (10.8%)	4 (12.1%)
Ex-smoker, n (%)	14 (37.8%)	14 (42.4%)
Pack years, py	14.4 ± 18.5	14.9 ± 19.6
Hypertension, n (%)	19 (51.4%)	20 (60.6%)
Diabetes, n (%)	9 (24.3%)	9 (27.3%)
Coronary artery disease, n (%)	7 (18.9%)	3 (9.1%)
Heart failure, n (%)	1 (2.7%)	0 (0.0%)
Dyslipidemia, n (%)	10 (27.0%)	7 (21.2%)
Obesity, n (%)	22 (59.5%)	24 (72.7%)
**OSA characteristics**		
OSA diagnosis since, years	8.4 ± 4.1	7.2 ± 5.4
AHI at time of diagnosis, events/h	51.8 ± 20.0	50.6 ± 24.9
ODI at time of diagnosis, events/h	50.0 ± 19.3	49.4 ± 25.1
Epworth Sleepiness Scale, points	7.0 ± 3.5	7.5 ± 3.4
CPAP usage, % days of last year	94 ± 7	93 ± 8
Residual AHI under CPAP therapy, events/h		
**Sex hormone levels at baseline**	2.9 ± 2.6	2.6 ± 1.9
17OH-Progesterone, ng/mL	0.61 (0.49, 1.17)	0.74 (0.36, 1.19)
Progesterone, ng/mL	0.03 (0.01, 0.09)	0.03 (0.02, 0.10)
Testosterone, ng/mL	3.42 (2.22, 4.18)	3.10 (1.61, 4.19)
Pregnenolone, ng/mL	1.18 (0.42, 2.16)	1.11 (0.74, 2.06)
Testosterone sum, nmol/L	10 (8, 15)	5 (1, 15)
Free testosterone, pg/mL	17 (14, 23)	12 (4, 19)
FSH, U/L	9 (4, 14)	9 (4, 44)
LH, U/L	5 (3, 8)	6 (3, 21)
Dehydroepiandrosterone, ng/mL	2.11 (1.42, 4.42)	2.1 (1.4, 3.8)
Androstenedione, ng/mL	0.72 (0.52, 1.06)	0.70 (0.56, 1.05)

Data are mean ± SD, median (IQR), or n (%), as appropriate. AHI, apnoea–hypopnoea index; BMI, body mass index; CPAP, continuous positive airway pressure; ODI, oxygen desaturation index; OSA, obstructive sleep apnoea.

**Fig. 1:**
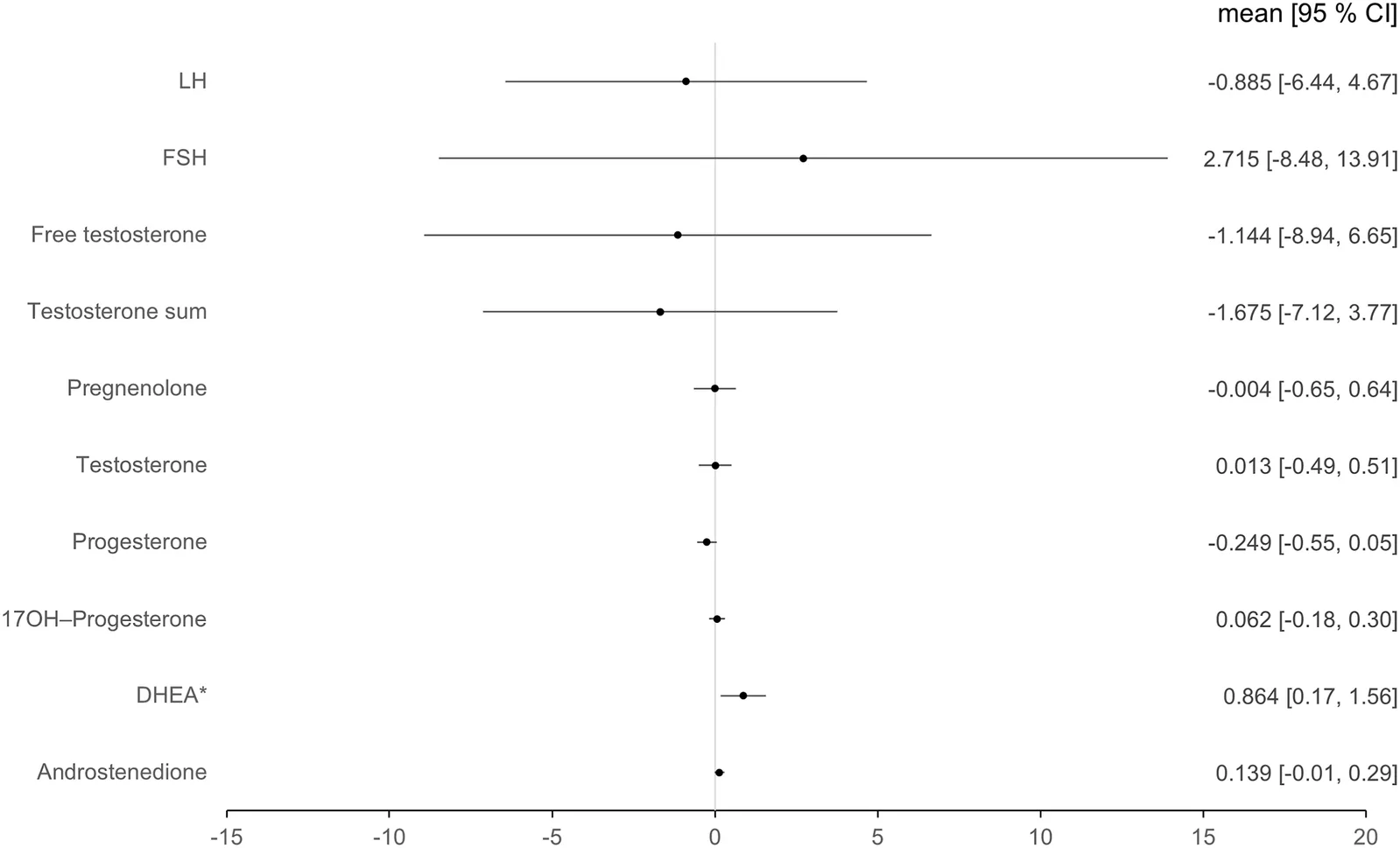
Effects of CPAP withdrawal for the secondary endocrine outcomes after multiple imputation, adjusted for the corresponding baseline hormone level, parent study, sex, age, BMI, and the presence of hypertension ([ng/ml] for steroid hormones, [U/l] for FSH and LH]).

The effectiveness of the CPAP withdrawal in terms of a recurrence of OSA was suggested by a significant increase in AHI (mean change AHI, 31.8 events/h, 95% CI + 22.8 to +40.6, p < 0.001) and ODI (mean change ODI, 31.5 events/h, 95% CI + 22.4 to +40.5, p < 0.001). More details are documented in an online supplementary file [5]. Baseline Epworth Sleepiness Scale (ESS) scores in the pooled cohort were 7.0 ± 3.5 in the therapeutic CPAP group and 7.5 ± 3.4 in the CPAP-withdrawal group (Table 1). In Thiel et al. CPAP withdrawal increased ESS by 3.29 points (95% CI 0.87 to 5.72; p = 0.009) [3]. Schwarz et al. reported ESS under established therapeutic CPAP (5.1 ± 2.2 and 6.5 ± 2.5 points in the subsequently assigned therapeutic CPAP and CPAP-withdrawal groups, respectively) [4]; a longitudinal ESS treatment effect during the randomised withdrawal period was not reported in that publication.

## 4 Discussion

In line with the results of a prior meta-analysis [6], which included two randomised controlled trials, we observed no statistically significant effect of CPAP withdrawal on testosterone levels. Estradiol was not assessed in the present study. Previous reports have similarly shown no consistent effect of CPAP therapy on estradiol or testosterone concentrations [7]. Nevertheless, the beneficial effects of CPAP on sexual quality of life [8] raise the possibility that sex hormones other than estradiol and testosterone may contribute to sexual dysfunction in OSA.

DHEA, a weak androgen and major precursor of sex steroid synthesis, is of particular interest in this context. Decreased levels of DHEA have been linked with SD, and DHEA replacement has been shown to improve sexual desire in both women and men [9]. Therefore, our a priori hypothesis would support an increase in DHEA levels in adherent CPAP users and therefore a decrease with CPAP withdrawal. Our analysis, however, showed a contrary result of CPAP withdrawal on DHEA levels. One possible explanation is that DHEA secretion is directly stimulated by adrenocorticotropic hormone and may therefore be regarded as a marker of endocrine stress response. Psychological stress and its associated neuroendocrine dysregulation are recognized risk factors for sexual dysfunction [10].

In this context, our findings argue against a direct role of sex hormone receptor–mediated pathways in OSA-associated SD and instead point towards predominantly autonomic and stress-mediated pathways, likely related to sympathetic activation, stress hormones, and oxidative stress.

The study cohort was predominantly male, reflecting the sex distribution of many clinical CPAP-treated OSA cohorts. As this analysis was not designed or powered to assess sex-specific endocrine responses, no subgroup analyses were performed. However, the findings should be interpreted primarily in the context of a predominantly male CPAP-treated OSA population, and generalizability to women is limited. Furthermore, free testosterone, testosterone sum, FSH and LH were not measured in one parent trial; the resulting design-based missingness limits the robustness of these estimates, which should be considered exploratory. Although both parent trials used the same 2-week withdrawal paradigm and study origin was included in the models, residual between-study heterogeneity cannot be completely excluded. The precise kinetics of individual sex-hormone responses over a 2-week withdrawal period also remain incompletely defined.

Short-term CPAP withdrawal did not significantly affect serum testosterone or most other sex hormones in patients with OSA. The observed increase in DHEA might reflect a short-term endocrine stress response. Studies with longer follow-up and validated assessments of sexual function are warranted to establish the clinical significance of these findings.

## CRediT authorship contribution statement

**Fabian A. Grewe:** Writing – review & editing, Writing – original draft, Methodology, Investigation, Formal analysis, Conceptualization. **Sira Thiel:** Methodology, Investigation, Funding acquisition, Formal analysis, Data curation, Conceptualization. **Sarah R. Haile:** Data curation. **Mirko Peitzsch:** Investigation. **Esther I. Schwarz:** Methodology, Investigation, Funding acquisition, Data curation. **Noriane A. Sievi:** Methodology, Investigation. **Felix Beuschlein:** Methodology, Investigation. **Malcolm Kohler:** Resources, Project administration. **Thomas Gaisl:** Supervision, Project administration, Investigation, Funding acquisition, Data curation, Conceptualization.

## Funding

The trial performed by *Schwarz* et al. was supported by grants from the 10.13039/100000001Swiss National Science Foundation (CR23I2_149617 and 32003B_143365/1), the 10.13039/501100006447University of Zurich Clinical Research Priority Program Sleep and Health and a Marie Curie European Reintegration Grant within the 7th European Community Framework Programme (276860).

## Declaration of competing interest

MK reports grants from University of Zurich, grants from Lunge Zurich, during the conduct of the study; grants from Roche Diagnostics (consultancy), outside the submitted work. TG reports grants from Bayer AG (consultancy), outside the submitted work. All other authors have nothing to declare.
