# Supplementary material for: Effects of CPAP withdrawal on sex hormone levels in patients with obstructive sleep apnoea: data from two randomised, controlled trials
Source: Sleep Med X. 2026 Sep 11;12:100201. doi: 10.1016/j.sleepx.2026.100201 (PMC13625797; doi:10.1016/j.sleepx.2026.100201)
Supplement: Supplementary file 1 [file mmc1.docx]

| **Hormone** | **Available** | **Missing** |
| --- | --- | --- |
| DHEA | 67 | 3 |
| DHEA-S | 67 | 3 |
| 17-OH-Progesterone | 67 | 3 |
| Androstenedione | 67 | 3 |
| Progesterone | 57 | 13 |
| Testosterone | 67 | 3 |
| Pregnenolone | 50 | 20 |
| Testosterone sum | 23 | 47 |
| Free testosterone | 23 | 47 |
| FSH | 23 | 47 |
| LH | 23 | 47 |

**Supplementary table 1 – Missing values**

Supplementary Table 1 shows the numbers of missing values for each of the measured sex hormones.
